# Delta/Notch-Like EGF-Related Receptor (DNER) Is Not a Notch Ligand

**DOI:** 10.1371/journal.pone.0161157

**Published:** 2016-09-13

**Authors:** Maxwell Greene, Yongjie Lai, Kostandin Pajcini, Will Bailis, Warren S. Pear, Eric Lancaster

**Affiliations:** 1 Department of Neurology, University of Pennsylvania, Perelman School of Medicine, Philadelphia, United States of America; 2 Department of Pathology and Laboratory Medicine, University of Pennsylvania, Perelman School of Medicine, Philadelphia, United States of America; 3 Department of Pharmacology, University of Illinois, Chicago, United States of America; 4 Department of Immunobiology, Yale School of Medicine, New Haven, Connecticut, United States of America; Institut Pasteur, FRANCE

## Abstract

Delta/Notch-like EGF-related receptor (DNER) has been reported to act as a Notch ligand, despite lacking a Delta/Serrate/Lag (DSL) binding domain common to all other known ligands. The established Notch ligand Delta-like 1 (DLL1), but not DNER, activated Notch1 in a luciferase assay, prevented the differentiation of myoblasts through Notch signaling, and bound Notch-fc in a cell-based assay. DNER is not a Notch ligand and its true function remains unknown.

## Introduction

Delta/Notch-like EGF-related receptor (DNER) is a transmembrane protein primarily found in the central nervous system (CNS) and specifically, found abundantly in Purkinje neurons of the cerebellum[1]. Genetic deletion of DNER in mice results in cerebellar ataxia, gross cerebellar abnormalities and microscopic abnormalities in Purkinje cell morphology[2]. DNER has recently been shown to be the autoantigen of a form of paraneoplastic cerebellar degeneration previously called “anti-Tr”[3], a finding that has been independently confirmed[4].

Notch signaling appears widely in metazoans to determine fate between cell populations[5], where classical Notch signaling involves a cell presenting Notch to an apposed cell presenting a Notch ligand (*trans* configuration) such as Delta-like 1 (DLL1). Although there is some homology to canonical Notch ligands, DNER lacks the Delta/Serrate/Lag (DSL) binding domain [6], which is thought to be essential for Notch ligands[7, 8]. Despite this structural difference, one heavily cited paper offered several lines of evidence to demonstrate that DNER functions as a Notch ligand[6], but this finding has not been replicated in the literature. The lines of evidence include co-culture luciferase assays, a C2C12 cell (myoblast) differentiation assay, and cell surface binding assays[6]. C2C12 cells have been widely used as a model system for canonical Notch signaling. C2C12 myoblasts have high levels of endogenous Notch, which, if activated, prevents their differentiation to myotubes[9–14]. In the original DNER differentiation experiments, the authors claim that myoblast differentiation could be demonstrated after 24 hours[6], a surprising result as the formation of myotubes has been shown to require between 2 and 6 days in low serum differentiation medium[9–14]. Here, we have performed co-culture luciferase assays, a C2C12 cell (myoblast) differentiation assay, and cell surface binding assays to determine whether DNER is a functional Notch ligand like DLL1.

## Materials and Methods

### Cell culture and transfection

Briefly, cultured cells (HEK293T, U2OS, and C2C12; ATCC, Virginia, USA) were grown in either 6 cm or 10 cm plates with growth media (Dulbecco’s Modified Eagle’s Medium (DMEM) (Life Technologies), 10% fetal bovine serum (FBS) (Gibco), 1% Anti-Anti (Life Technologies), or 1% penicillin/streptomycin, and L-glutamine (Life Technologies)). Cells were split when grown to 70% confluence with either 0.05% trypsin (Invitrogen Cat#25300054) or 0.25% trypsin (Invitrogen Cat#25200056) for HEK293T and U2OS cells, and at 50% confluence for C2C12 cells. Experiments were carried out, unless otherwise noted, with cells grown to 70% confluence.

### Luciferase assay for Notch1 signaling

A luciferase assay was used to demonstrate Notch activity. Transient transfection of cultured cells was accomplished with the Fugene/Optimem (Promega) system and according to the manufacturer’s instructions with the following plasmids at a concentration, unless otherwise stated, of 0.1 μg/well of DNA per plasmid of a 96-well plate: DNER (generous gift of Drs. De Graaff and Sillevis-Smitt, Erasmus Medical Center, Rotterdam the Netherlands, originally from M. Kengaku), DLL1 (EX-Y3540-M11, GeneCopoeia, Rockville, MD, USA), empty vector on a p-receiver backbone (GeneCopoeia, Rockville, MD, USA), *Renilla* (luciferase control, 0.01 μg/well, pRL-TK, Promega E2241), TP1 (very sensitive intracellular Notch reporter with twelve CSL binding sites converting intracellular Notch activity to firefly luminescence, which is capable of detecting slight perturbations in signaling strength with little background), and full length human Notch1 on a MigR1 backbone (both generous gifts of Dr. Jon Aster).

A *cis* luciferase assay was constructed as follows: 10,000 U2OS cells per well (200,000 cells/mL, 50 μL per well) were transiently transfected with ligand (DLL1, DNER, or empty vector), full-length Notch1, TP1 (the reporter of Notch activity), and the control luciferase *Renilla* in wells of a white 96-well plate. Each condition was incubated with either γ-secretase inhibitor (GSI) 1 μM/well (compound E XXI, cat# 565790-1mg, from EMD millipore), or sterile DMSO 1 μM/well (+GSI or -GSI). Each experimental run was performed in triplicate. After 48–72 hours of culture to allow for Notch signaling, britelite (Perkin Elmer) and Stop and Glo (Promega) were used to activate luminescence, and the luminescence was read using a Glomax 96 micro plate luminometer (Promega). Firefly luminescence was normalized to *Renilla*. Two separate experiments, each performed with repeats of 3–4 wells per condition were combined and normalized to empty vector in each experiment. For DLL1, DLL1 +GSI, DNER, DNER +GSI, empty vector, and empty vector +GSI, total n = 8, 8, 8, 8, 8, and 6, respectively.

A *trans* luciferase assay was constructed as follows: 10,000 U2OS cells per well (200,000 cells/mL, 50 μL per well) were transiently transfected with full-length Notch1, TP1, and *Renilla* in wells of a white 96-well plate. Each condition was incubated with either GSI 1 μM/well (+GSI), or sterile DMSO 1 μM/well (-GSI). Separately, other U2OS cells (200,000 cells/mL) were transiently transfected with plasmids causing the expression of ligand (DLL1, DNER, or empty vector) in 24-well plates (0.5 μg of DNA/well), and after 24 hours of separate growth and expression, the cells containing ligand were trypsinized and split together with the cells expressing Notch1/TP1/*Renilla* on the 96 well plate. Each experimental run was performed in triplicate. After 24–48 hours of co-culture to allow for Notch signaling, britelite and Stop and Glo were used to activate luminescence, and the luminescence was read using a Glomax 96 micro plate luminometer. Firefly luminescence was normalized to *Renilla*. Four separate experiments, each performed with repeats of 2–4 wells per condition were combined and normalized to empty vector in each experiment. For DLL1, DLL1 +GSI, DNER, DNER +GSI, empty vector, and empty vector +GSI, total n = 15, 15, 14, 13, 15, and 12, respectively.

The results were analyzed using an ordinary one-way ANOVA with multiple comparisons.

### C2C12 myoblast differentiation experiments

Since Notch is thought to more effectively bind its ligands when clustered[15], pre-clustered DNER-fc and DLL1-fc were used. To create pre-clustered DNER-fc and DLL1-fc, the -fc chimeras (5 μg/mL) were treated with 3 different concentrations of secondary antibodies (1, 5, or 50 μg/mL). Rabbit anti-human-fc (Jackson ImmunoResearch Laboratories, Inc, West Grove, PA, USA) was combined with DNER-fc (2254-DN-050 R&D Systems, Minneapolis, MN, USA) at the above concentrations in differentiation media (2% horse serum, Invitrogen cat#16050122), and kept at RT for 1 hour. Rabbit anti-mouse-fc (Jackson ImmunoResearch Laboratories, Inc, West Grove, PA, USA) was combined with DLL1-fc (3970-DL-050 R&D Systems, Minneapolis, MN, USA) the above concentrations in media (2% horse serum), and kept at RT for 1 hour.

C2C12 cells were grown to 50% confluence and split together with differentiating media (2% horse serum) that contained either pre-clustered DNER-fc or pre-clustered DLL1-fc at the different concentrations described previously, unclustered DNER-fc, or rabbit anti-human-fc (-fc only). C2C12 cells mixed with the mixture of differentiation media and -fc chimeric antibodies were allowed to incubate at 37°C in an incubator (5% CO2) for 72 hours and cells were fixed, and levels of myotube formation were assessed. Myotubes were visualized using antibody to myosin sarcomere (1:200) to label for fusion (DSHB product MF20, University of Iowa, USA) and stained with a TRITC secondary antibody (1:200). Cells were visualized with an inverted microscope, and were assessed for nuclei in myotubes vs. nuclei outside of myotubes (fusion index) by two independent investigators, one blinded. Results were further analyzed with an ordinary one-way ANOVA with multiple comparisons.

### Binding of Notch-fc to transfected cells

U2OS cells were transiently transfected with plasmids to express either DNER or DLL1, and cultured for 48 hours to allow for expression. Notch-fc chimera (1057-tk R&D Systems, Minneapolis, MN, USA) (5 μg/mL and 25 μg/mL) was pre-clustered with mouse anti-human-fc (Jackson ImmunoResearch Laboratories, Inc, West Grove, PA, USA) (5 μg/mL and 50 μg/mL) in cell culture media for 1 hour at 37°C. The pre-clustered Notch-fc was then incubated with the transiently transfected cells for 1 hour at 37°C in an incubator with 5% CO2. Treated cells were then washed 3 times with PBS, fixed for 5 minutes with 4% paraformaldehyde, washed 3 times with PBS, permeabilized for 5 minutes with 0.3% Triton X-100 in PBS, washed 3 times with PBS, and blocked with 4% bovine serum albumin (BSA) for 1 hour. Specific antibodies for DNER (R&D Systems AF2254) or DLL1 (Abcam ab10554) were applied for 1 hour at room temperature, followed by appropriate secondary fluorescent antibodies to visualize the expression of DNER, DLL1 and Notch-fc, and then mounted with fluoromount-G with DAPI (OB010020, SouthernBiotech, Birmingham, AL, USA).

## Results

**Fig 1:**
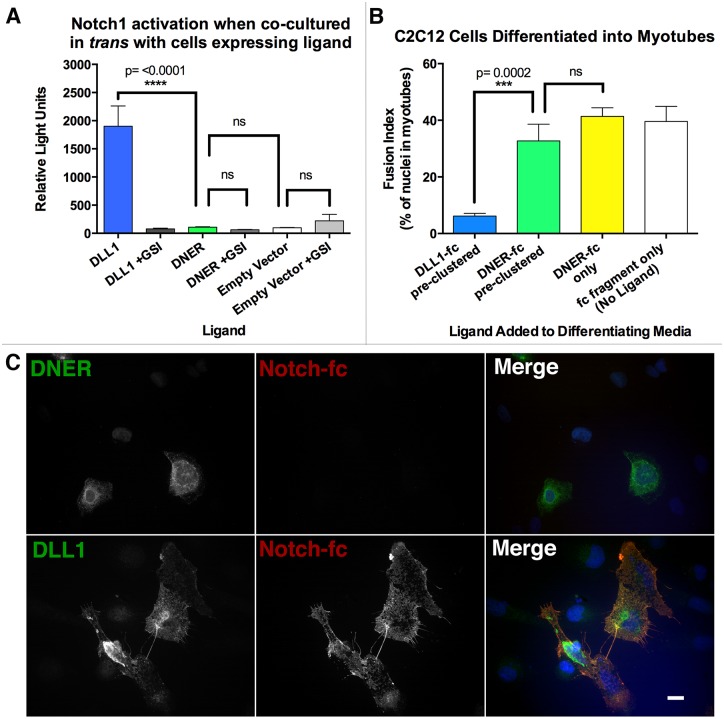
DNER does not activate, function with, or bind to Notch, but known Notch ligand DLL1 does. (A) Pooled luciferase results from 4 separate experiments (normalized to the mean of empty vector in each experiment). U2OS cells were transfected with ligand (DLL1, DNER, or EV), and separately a population of U2OS cells was transfected to express Notch, the control luciferase *Renilla*, and TP1, a promoter that expresses firefly luciferase when Notch is activated. The two populations were co-cultured 24 hours after transfection (*trans* configuration), and activity read after an additional 24–48 hours of incubation. (B) C2C12 cells (myoblasts) were incubated with differentiation media (2% horse serum) that either had pre-clustered DLL1-fc (1:1), pre-clustered DNER-fc (1:1), un-clustered DNER-fc, or fc only, all at a ratio of 1:150 in media. Cells were incubated for 72 hours, then fixed, and stained for the presence of myosin heavy chain (MHC) and nuclei. By measuring the percent of total nuclei that were inside of differentiated MHC positive myotubes, fusion indexes were calculated. (C) DNER (top left, green) transfected U2OS cells were not labeled by pre-clustered Notch-fc (top middle, red) but DLL1 (bottom left, green) transfected U2OS cells were labeled by pre-clustered Notch-fc (bottom middle, red). Merged images are shown at far right. Scale 10 μM. **** = p value <0.0001. *** = p value 0.002. ns = not significant. DLL1 = Delta-like 1, a known Notch Ligand, DNER = Delta/Notch-like epidermal growth factor (EGF) related receptor, GSI = γ-secretase inhibitor, fc only = rabbit anti-human-fc.

To assess DNER’s capacity to function as a Notch ligand, we repeated the key experiments supporting this conclusion. U2OS cells were transfected to express Notch1 and a luciferase-based reporter of Notch signaling (Fig 1A). We used a sensitive reporter with twelve CSL binding sites, which is capable of detecting slight perturbations in Notch1 signaling strength with little background. These transfected cells showed a robust luminescent signal (p<0.0001 compared with controls) when mixed in *trans* with U2OS cells expressing DLL1. This signal was eliminated in the presence of γ-secretase inhibitor (GSI), indicating that it depends on classical Notch signaling[16]. However, U2OS cells transiently transfected in an identical manner showed no significant change in signal above background when exposed in *trans* to cells expressing DNER. A weaker effect was also seen when DLL1 was in *cis* with Notch. While DLL1 showed significant *cis* activation of Notch (p = 0.0003 compared with empty vector and p = <0.0001 when compared with DNER) that was eliminated with GSI, DNER did not show significant *cis* Notch activation when compared with controls (S1 Fig). To verify that the DNER and DLL1 constructs were successfully expressed, some cells were immunostained for DNER or DLL1; both constructs were expressed in the transfected cells (data not shown).

We next tested the ability of DNER-fc to prevent differentiation of C2C12 cells into myotubes. DLL1-fc and DNER-fc were separately pre-clustered, mixed with differentiation media, and then added to cultured C2C12 cells. Three days later cells were fixed and fusion indexes were measured. Pre-clustered DLL1-fc significantly (p = 0.0002) reduced the formation of myotubes when compared to either DNER-fc treated populations or untreated controls (Fig 1B). In contrast, DNER-fc did not significantly prevent differentiation when compared to controls, regardless of clustering.

Finally, the ability of Notch1 to bind to DLL1 or to DNER was tested using a cell-based model system. Notch1-fc was pre-clustered with an anti-mouse-fc and then added to living U2OS cells transiently transfected to express either DLL1 or DNER. We next verified that DNER is expressed on the membrane and accessible to ligands in the culture media, as previously reported for HEK cells[4]. U2OS cells transfected to express DNER were live-stained with a DNER antibody targeting an extracellular epitope, and showed punctate staining of DNER on the plasma membrane of transfected cells, while untransfected cells were unstained (S2 Fig). Pre-clustered Notch-fc bound to the membrane of cells transfected with DLL1, but did not bind to cells transfected with DNER (Fig 1C).

## Conclusion

Collectively, these data suggest that DNER does not function as a Notch ligand. DNER does not induce Notch activation in a luciferase assay in contrast to the classical ligand DLL1, is unable to prevent the differentiation of cultured myoblasts like DLL1, and is unable to bind pre-clustered Notch-fc like DLL1. DNER therefore does not appear to be a ligand for the Notch1 receptor. While DNER has some homology to Notch ligands, the absence of a DSL binding domain in DNER may be the crucial factor that prevents it from binding to or activating Notch in the manner of the well-established and canonical Notch ligands.

The profound cerebellar dysfunction associated with genetic deletion of DNER[2] and separately with paraneoplastic autoantibodies to DNER[3, 4], suggest not only that it plays a role during development, but that it may have persistent functions in the adult cerebellum, however this is currently unclear. Further investigation is needed to establish the true binding partners and functions of this important cerebellar protein.

## Supporting Information

S1 Fig DNER does not significantly activate Notch in *cis* while DLL1 does. Pooled Luciferase results from 2 separate experiments (normalized to the mean of empty vector in each experiment). U2OS cells were transfected with ligand (DLL1, DNER, or EV), Notch, the control luciferase *Renilla*, and TP1, a promoter that expresses firefly luciferase when Notch is activated. Notch activity was read after 48–72 hours of incubation. DLL1 shows Notch activation in *cis*, which is eliminated with GSI, while DNER with or without GSI does not significantly activate Notch when compared with empty vector. **** = p value <0.0001. ns = not significant. DLL1 = Delta-like 1, a known Notch Ligand, DNER = Delta/Notch-like epidermal growth factor (EGF) related receptor, GSI = γ-secretase inhibitor.
(TIF)

S2 Fig Transfected U2OS cells that express DNER display DNER at the plasma membrane. DNER transfected U2OS cells were live-stained for 1 hour for the presence of DNER. Transfected cells were labeled in a punctate manner (DNER labeled in green). Scale 10 μM.
(TIF)

## Abbreviations

In Text γ: Gamma
