# Supplementary figures and images for: Delta/Notch-Like EGF-Related Receptor (DNER) Is Not a Notch Ligand
Source: PLoS One. 2016 Sep 13;11(9):e0161157. doi: 10.1371/journal.pone.0161157 (PMC5021350; doi:10.1371/journal.pone.0161157)

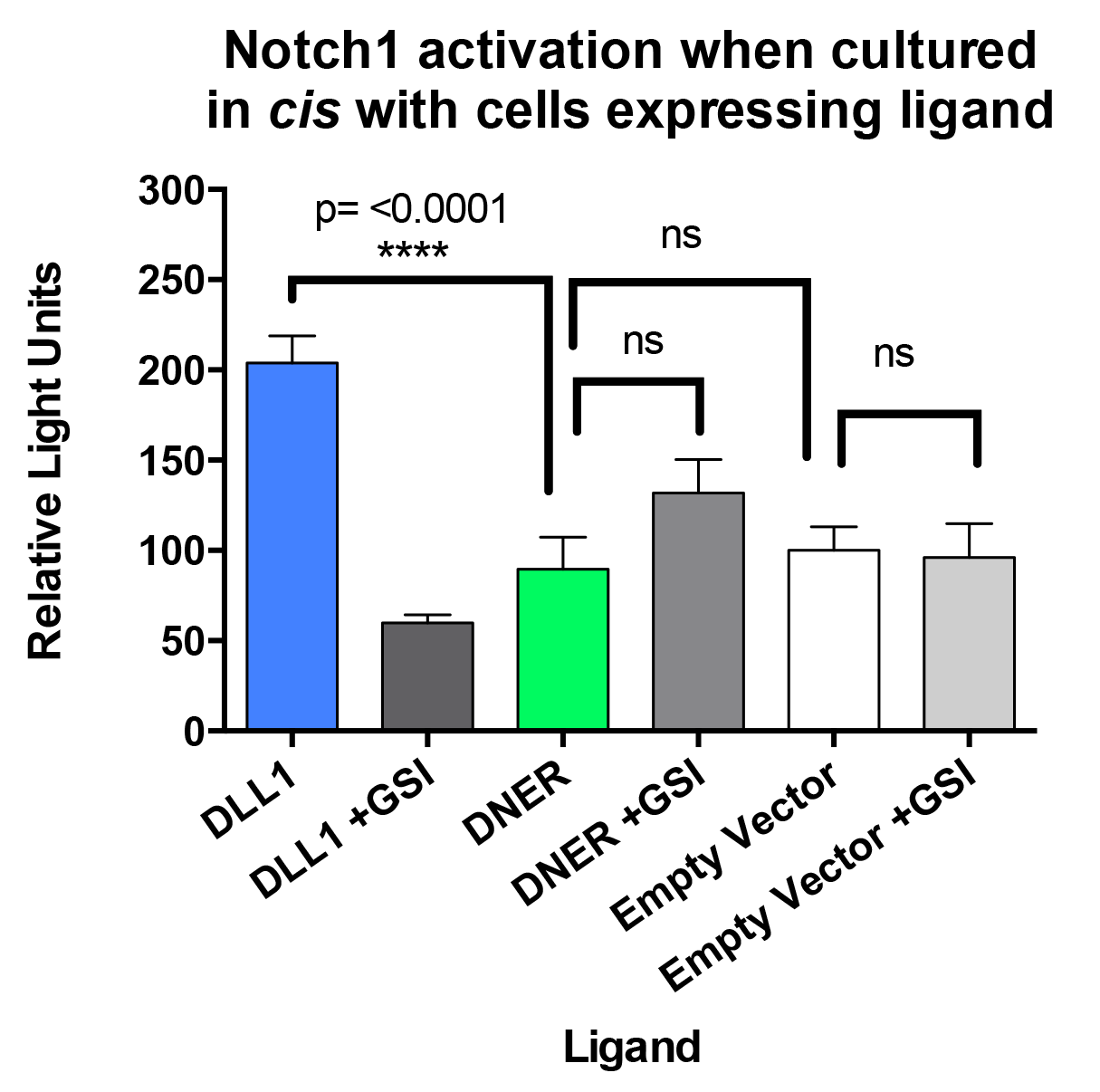

Supplement: S1 Fig — Pooled Luciferase results from 2 separate experiments (normalized to the mean of empty vector in each experiment). U2OS cells were transfected with ligand (DLL1, DNER, or EV), Notch, the control luciferase Renilla, and TP1, a promoter that expresses firefly luciferase when Notch is activated. Notch activity was read after 48–72 hours of incubation. DLL1 shows Notch activation in cis, which is eliminated with GSI, while DNER with or without GSI does not significantly activate Notch when compared with empty vector. **** = p value <0.0001. ns = not significant. DLL1 = Delta-like 1, a known Notch Ligand, DNER = Delta/Notch-like epidermal growth factor (EGF) related receptor, GSI = γ-secretase inhibitor. (TIF) [file pone.0161157.s001.tif]

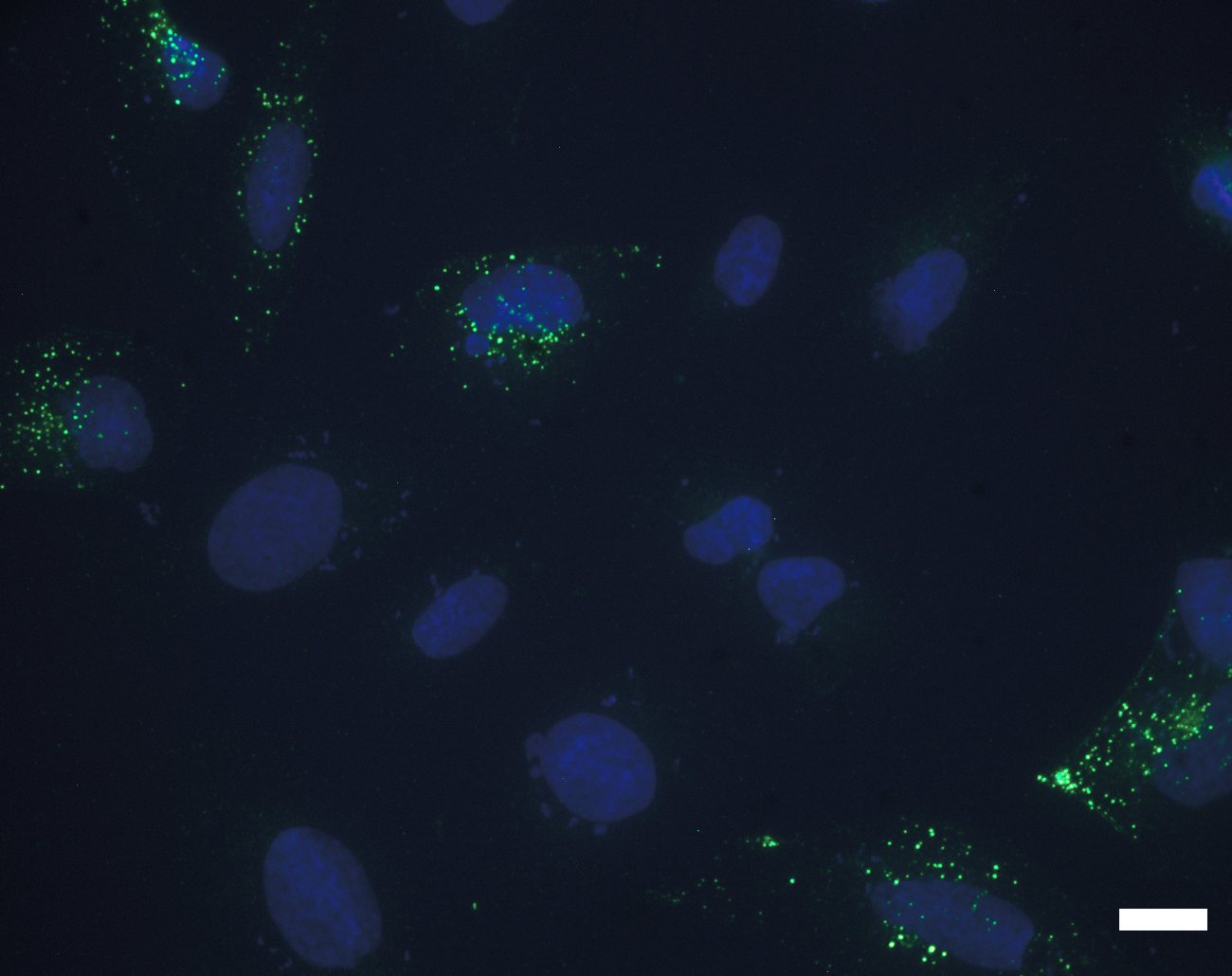

Supplement: S2 Fig — DNER transfected U2OS cells were live-stained for 1 hour for the presence of DNER. Transfected cells were labeled in a punctate manner (DNER labeled in green). Scale 10 μM. (TIF) [file pone.0161157.s002.tif]
